# AMPK phosphorylates NAMPT to regulate NAD^+^ homeostasis under ionizing radiation

**DOI:** 10.1098/rsob.220213

**Published:** 2022-10-05

**Authors:** Xiaoyu Liao, Xiaoke Huang, Xin Li, Xuemei Qiu, Mi Li, Rui Liu, Tao He, Qingfeng Tang

**Affiliations:** ^1^ State Key Laboratory of Oral Diseases, National Clinical Research Center for Oral Diseases, Research Unit of Oral Carcinogenesis and Management, Chinese Academy of Medical Sciences, West China Hospital of Stomatology, Sichuan University, Chengdu, Sichuan 610041, People's Republic of China; ^2^ Department of Urology, Xindu district People's hospital of Chengdu, Chengdu, Sichuan 610500, People's Republic of China; ^3^ UTHealth Graduate School of Biomedical Sciences, Houston, TX 77225, USA; ^4^ Department of cardio-thoracic Surgery, The Second Affiliated Hospital of Chengdu Medical College, China National Nuclear Corporation 416 Hospital, Chengdu, Sichuan, People's Republic of China

**Keywords:** NAMPT, AMPK, NAD^+^, ionizing radiation, phosphorylation

## Abstract

Radiation-induced oral mucositis is the most common complication for patients who receive head/neck radiotherapy. Nicotinamide adenine dinucleotide (NAD^+^) is vital for DNA damage repair under ionizing radiation, through functioning as either the substrate for protein poly(ADP-ribosyl)ation at DNA break sites or the cofactor for multiple DNA repair-related enzymes, which therefore can result in a significant consumption of cellular NAD^+^ during DNA repair. Mammalian cells produce NAD^+^ mainly by recycling nicotinamide via the salvage pathway, in which the rate-limiting step is governed by nicotinamide phosphoribosyltransferase (NAMPT). However, whether NAMPT is co-opted under ionizing radiation to timely fine-tune NAD^+^ homeostasis remains elusive. Here we show that ionizing radiation evokes NAMPT activation within 30 min without apparent changes in its protein expression. AMPK rapidly phosphorylates NAMPT at S314 under ionizing radiation, which reinforces the enzymatic activity of NAMPT by increasing NAMPT binding with its substrate phosphoribosyl pyrophosphate (PRPP). AMPK-mediated NAMPT S314 phosphorylation substantially restores NAD^+^ level in the irradiated cells and facilitates DNA repair and cell viability. Our findings demonstrate a new post-translational modification-based signalling route, by which cells can rapidly orchestrate NAD^+^ metabolism to support DNA repair, thereby highlighting NAMPT as a potential target for the prevention of ionizing radiation-induced injuries.

## Graphical abstract

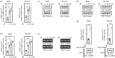

## 1. Introduction

Nicotinamide adenine dinucleotide (NAD^+^) is widely established as an essential cofactor for electron transfer functioning in diverse metabolic pathways [1]. Particularly, NAD^+^ plays critical roles in glycolysis in cytosol and the tricarboxylic acid cycle in mitochondria where it generates reducing force in the form of NADH, which then transfers electrons from various sources to the mitochondrial complex I and downstream components of the electron transport chain, ultimately leading to the production of ATP [2].

NAD^+^ can be synthesized through the *de novo* pathway from tryptophan, the Preiss-handler pathway from nicotinic acid, or the salvage pathway by recycling of nicotinamide, and the latter is the major route for NAD^+^ biosynthesis for mammalians [3]. The rate-limiting step of salvage pathway, by which nicotinamide and PRPP were condensated to generate nicotinamide mononucleotide (NMN), is catalysed by nicotinamide phosphoribosyltransferase (NAMPT) [4]. NAD^+^ is eventualy produced from NMN in mammalian cells by three nicotinamide mononucleotide adenylyltransferases (NMNATs), with NMNAT1 located in the nucleus, NMNAT2 located in the Golgi apparatus, and NMNAT3 located in mitochondria [5].

In addition to being an electron carrier, NAD^+^ also function as the substrate for protein poly(ADP-ribosyl)ation, by which polymers of ADP-ribose are covalently linked to proteins through poly (ADP-ribose) polymerases (PARPs) [6]. Poly(ADP-ribosyl)ation has significant impacts on the cellular responses to DNA strand breaks under ionizing radiation [7]. As early DNA damage sensors, PARPs are rapidly activated by binding with the ionizing radiation-elicited DNA breaks, and catalyse poly(ADP-ribosyl)ation on itself as well as adjacent histones and other proteins, thereby marking the DNA lesion sites along the chromatin. The poly(ADP-ribose) chain of the modified proteins can then recruit other effector proteins to the lesion site and locally assemble the DNA damage-responsive complexes, thereby promoting chromatin relaxation and initiating DNA repair process [8].

Radiation-induced oral mucositis (RIOM) is the most common complication for patients who receive head and neck radiotherapy, which may cause multiple temporary or irreversible damages in oral mucosa [9]. RIOM-mediated inflammation, which may lead to ulceration, is highly toxic to epithelial and endothelial cells in oral mucosa [10]. During the repair of ionizing radiation-induced DNA breaks, the length of poly(ADP-ribose) chains may attain a size of 200–300 residues, and their synthesis increases up to 500-fold owing to the activation of PARPs, which can result in rapid and significant consumption of cellular NAD^+^ [11,12]. Given the essential role of poly(ADP-ribosyl)ation in DNA repair, NAD^+^ availability becomes a critical factor that may modulate DNA repair capacity [13]. Addition of NAD^+^ largely promoted DNA repair capacity of soluble cell extracts on ionizing radiation or radiomimetic agents-treated DNA [14]. However, whether NAMPT, as the rate-limiting enzyme in the salvage pathway for NAD^+^ biosynthesis, is co-opted under ionizing radiation to timely fine-tune NAD^+^ homeostasis remains elusive. In this study, we demonstrate that AMPK rapidly phosphorylates NAMPT at Serine (S)314 in oral keratinocyte and endothelial cells under ionizing radiation, which reinforces the enzymatic activity of NAMPT by increasing its binding with PRPP. AMPK-mediated NAMPT S314 phosphorylation substantially restores NAD^+^ level in the irradiated cells and facilitates cell viability.

## 2. Results

### 2.1. Ionizing radiation evokes cellular NAMPT activity

**Figure 1.**
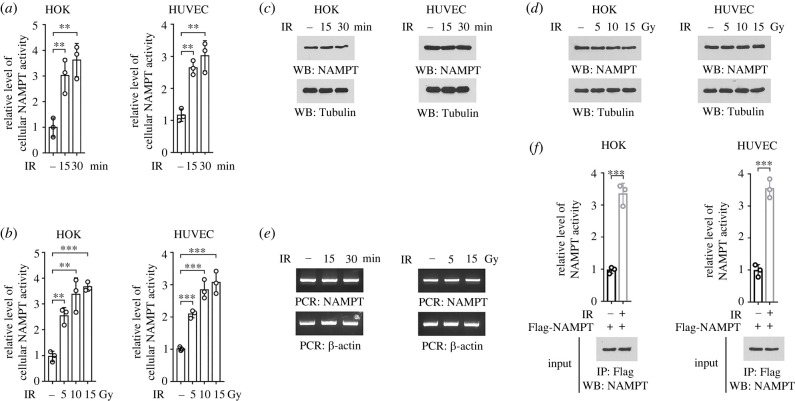
Ionizing radiation evokes cellular NAMPT activity. (*a,c*) HOK and HUVEC cells were treated with 10 Gy ionizing radiation, and cellular enzymatic activity of NAMPT was measured at indicated time after irradiation (*a*). The expression of NAMPT was examined by immunoblot (*c*). IR, ionizing radiation. **p < 0.01. (*b,d*) HOK and HUVEC cells were treated with ionizing radiation at indicated doses, and cellular enzymatic activity of NAMPT was measured 30 min after irradiation (*b*). The expression of NAMPT was examined by immunoblot (*d*). **p < 0.01; ***p < 0.001. (*e*) HOK cells were treated with ionizing radiation at 10 Gy (left panel) or indicated doses (right panel). The expression of NAMPT was examined by RT-PCR. (*f*) HOK and HUVEC cells with expression of Flag-NAMPT were treated with 10 Gy ionizing radiation, and Flag-NAMPT protein was precipitated. NAMPT activity in the precipitates was measured. ***p < 0.001.

To determine the impact of ionizing radiation on NAD synthesis, human oral keratinocytes (HOKs) and human umbilical vein endothelial cells (HUVECs) were used as *in vitro* models, since these cell types exist in oral mucosa and are reported to be sensitive to ionizing radiation [15,16]. Upon exposure to 10 Gy ionizing radiation, a 2.5–3.5-fold increase in the NAMPT enzymatic activity was observed in the lysates of HOK and HUVEC cells (figure 1*a*). The induction of cellular NAMPT activity was likely a rapid response to ionizing radiation, since it could be apparently detected as early as 15 min after irradiation. Further, similar effects could be observed in both HOK and HUVEC cells under radiation treatment at multiple doses (figure 1*b*).

Next, we moved to explore the underlying mechanism responsible for ionizing radiation-elicited cellular NAMPT activity, by measuring NAMPT expression at both protein and mRNA levels. Nevertheless, no obvious change was observed under multiple radiation treatments (figure 1*c–e*). Notably, a markedly higher enzymatic activity was detected in the Flag-NAMPT proteins precipitated from irradiated HOK or HUVEC cells, compared to that in equal amount of Flag-NAMPT protein derived from untreated counterpart cells (figure 1*f*). These results suggest that ionizing radiation evokes cellular NAMPT activity, which is independent of changes in NAMPT expression.

### 2.2. AMPK phosphorylates NAMPT S314 under ionizing radiation

**Figure 2.**
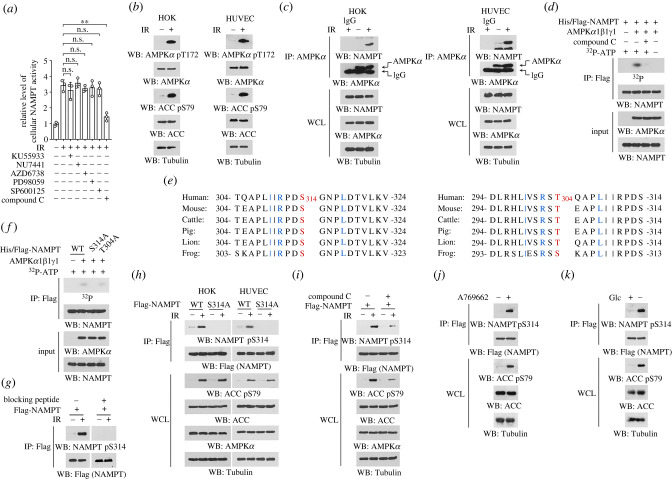
AMPK phosphorylates NAMPT S314 under ionizing radiation. (*b–d, f–k*) Immunoblotting analyses were performed using indicated antibodies. (*a*) HOK cells were pre-treated with 10 µM KU55933, 1 µM NU7441, 2 µM AZD6738, 20 µM PD98059, 20 µM SP600125 or 5 µM Compound C for 2 h, and cells were treated with 10 Gy ionizing radiation. Cellular NAMPT activity was measured 30 min after irradiation. **p < 0.01; ns, not significant. (*b,c*) HOK and HUVEC cells were treated with 10 Gy ionizing radiation, and cells were harvested 15 min after irradiation. Immunoblots (*b*) and immunoprecipitations (*c*) were performed using indicated antibodies. IR, ionizing radiation; WCL, whole cell lysate. (*d*) Bacterially purified His/Flag-NAMPT protein was incubated with purified active AMPK proteins (His-AMPK*α*1, untagged AMPK*β*1 and untagged AMPK*γ*1) in the presence or absence of Compound C and [γ-^32^P]-ATP for an *in vitro* kinase assay. Immunoprecipitation was performed using anti-Flag antibody, and radioactivity in the precipitates was measured by autoradiography. (*e*) Alignment analyses of NAMPT S314 or T304 was performed among indicated species. S314 and T304 were shown in red, and the residues matches AMPK phosphorylation consensus was shown in blue. (*f*) Bacterially purified WT His/Flag-NAMPT protein or indicated mutants were incubated with purified active AMPK proteins in the presence [γ-^32^P]-ATP for an *in vitro* kinase assay. Immunoprecipitation was performed using anti-Flag antibody, and radioactivity in the precipitates was measured by autoradiography. (*g*) HOK cells were treated with 10 Gy ionizing radiation. Immunoblots were performed using indicated antibodies in the presence or absence or NAMPT pS314 blocking peptide. (*h*) HOK and HUVEC cells with expression of WT Flag-NAMPT or Flag-NAMPT S314A were treated with 10 Gy ionizing radiation, and immunoprecipitation was performed 30 min after irradiation. (*i*) HOK cells with expression of Flag-NAMPT were pre-treated with 5 µM Compound C for 2 h, and treated with 10 Gy ionizing radiation. Immunoprecipitation was performed 30 min after irradiation. (*j*) HOK cells were treated with 0.5 mM A769662 for 30 min. (*k*) HOK cells were incubated with glucose-free medium for 12 h. Glc, glucose.

To determine the key factor that modulate NAMPT activity under ionizing radiation, HOK cells were pre-treated with small-molecular inhibitors to counteract a couple of radiation-responsive proteins. Treatment with Compound C, an AMPK inhibitor, substantially abolished ionizing radiation-induced NAMPT activity (figure 2*a*). By contrast, inhibition of ATM by KU55933, DNA-PK by NU7441, ATR by AZD6738, MEK/ERK pathway by PD98059 or JNK by SP600125 only showed minor effects (figure 2*a*). In addition to being an energy sensor, AMPK was found to be rapidly activated in response to ionizing radiation [17,18]. Indeed, apparent AMPK activation was observed in both HOK and HUVEC cells 15 min after exposure to ionizing radiation, reflected by the enhanced phosphorylation of AMPK*α* T172 and its substrate ACC S79 (figure 2*b*). Further, co-immunoprecipitation revealed much more NAMPT protein in the AMPK*α* precipitates derived from irradiated cells than that from untreated cells, suggesting that ionizing radiation cemented NAMPT binding with AMPK kinase (figure 2*c*).

AMPK functions as a stress sensor and signal transducer by phosphorylating diverse proteins [19], which prompted us to test whether NAMPT is a direct substrate for AMPK. We performed *in vitro* kinase assay by mixing the bacterially purified NAMPT protein with the purified active AMPK proteins (including subunit *α*1, *β*1 and *γ*1) in the presence of [γ-^32^P]-ATP. Notably, incubation with AMPK proteins resulted in an obvious band corresponding to NAMPT protein under autoradiography, indicating that the radioactive γ-phosphate from ATP was covalently linked to NAMPT protein by AMPK-mediated phosphorylation (figure 2*d*). This phosphorylation could be mostly abolished in the presence of Compound C, which rule out the possibility that NAMPT was phosphorylated by other unknown kinases contaminated during protein purification. The autoradiographic signal detected in NAMPT protein was not likely due to the previously reported ATP hydrolysis-mediated NAMPT autophosphorylation [20], since recombinate NAMPT protein was boiled before subjected to the reaction, and no detectable signal was found in NAMPT protein when AMPK proteins were not included in the reaction (figure 2*d*).

To identify the phosphorylation site, we analysed NAMPT protein sequence and found S314 site whose flanking sequence appropriately matched the AMPK substrate consensus L/IxRxx(pS/T)xxxL/I (figure 2*e*) [21]. In addition, analysis with SCANSITE 4.0 (https://scansite4.mit.edu/#home, Stringency: Low) revealed threonine (T)304 site as another putative AMPK phosphorylation site (figure 2*e*). Alignment comparison manifested that both two sites were evolutionally conserved among multiple species (figure 2*e*). Substitution of these sites into non-phosphorylatable alanine (A) showed that only S314 mutation annihilated AMPK-mediated phosphorylation (figure 2*f*). We thus generated an antibody recognizing NAMPT protein with phosphorylated S314, and, with this antibody, we found a sharply accumulated NAMPT S314 phosphorylation in the irradiated HOK cells. Detection of this phosphorylation could be mostly blocked if a NAMPT pS314 blocking peptide was used during immunoblotting (figure 2*g*), suggesting a good specificity of this antibody. Further, ionizing radiation-induced NAMPT S314 phosphorylation could be abrogated by either NAMPT S314 mutation, or Compound C treatment (figure 2*h–i*). As expected, accumulated NAMPT S314 phosphorylation was also found in non-irradiated cells that were treated with A769662 (figure 2*j*), a AMPK activator [22] or incubated with glucose-free medium (figure 2*k*). These results suggest that AMPK phosphorylates NAMPT S314 in the context of ionizing radiation.

### 2.3. AMPK-dependent S314 phosphorylation activates NAMPT by facilitating NAMPT binding with PRPP

**Figure 3.**
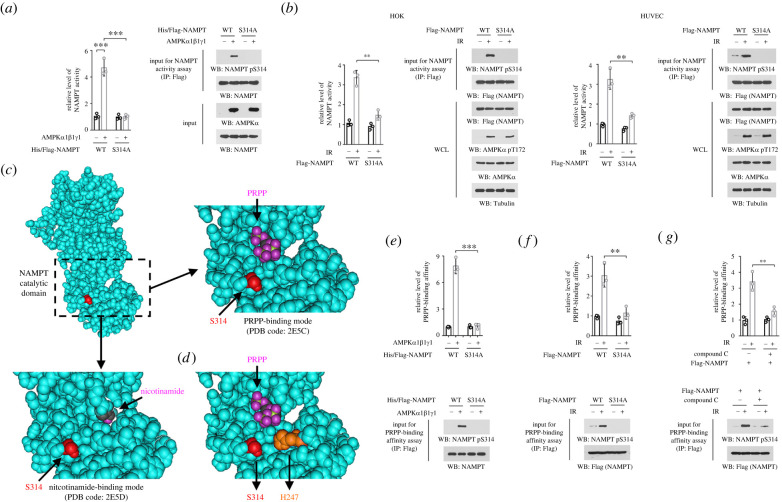
AMPK-dependent S314 phosphorylation activates NAMPT by facilitating NAMPT/PRPP association. (*a*) Bacterially purified unheated WT His/Flag-NAMPT or S314 mutant protein was incubated with purified active AMPK proteins for an *in vitro* kinase assay. His/Flag-NAMPT proteins were precipitated using anti-Flag antibody, and the NAMPT enzymatic activity in the precipitates was measured. ***p < 0.001. (*b*) HOK and HUVEC cells with expression of WT Flag-NAMPT or Flag-NAMPT S314A were treated with 10 Gy ionizing radiation. Flag-NAMPT proteins were precipitated from cell lysates 30 min after irradiation, washed twice with PBS, and the NAMPT enzymatic activity in the precipitates was measured. IR, ionizing radiation; WCL, whole cell lysate; **p < 0.01. (*c*) The NAMPT catalytic domain in human NAMPT protein (shown in cyan) structure was boxed by dotted line, and enlarged to show the spatial location of S314 (side chain shown in red), PRPP (oxygen atom shown in purple, PDB code: 2E5C), and nicotinamide (oxygen atom shown in purple, PDB code: 2E5D). (*d*) Human NAMPT protein structure (PDB code: 2E5C) shows the spatial location of S314 (side chain shown in red), PRPP (oxygen atom shown in purple), and H247 (side chain shown in orange). (*e*) Bacterially purified unheated WT His/Flag-NAMPT or S314 mutant protein was incubated with purified active AMPK proteins for an *in vitro* kinase assay. His/Flag-NAMPT proteins were precipitated using anti-Flag antibody, and the binding affinity between NAMPT protein and PRPP was measured. ***p < 0.001. (*f*) HOK cells with expression of WT Flag-NAMPT or Flag-NAMPT S314A were treated with 10 Gy ionizing radiation. Flag-NAMPT proteins were precipitated from cell lysates 30 min after irradiation, washed twice with PBS, and the binding affinity between NAMPT protein and PRPP was measured. **p < 0.01. (*g*) HOK cells with expression of WT Flag-NAMPT were pre-treated with 5 µM Compound C for 2 h, and cells were then treated with 10 Gy ionizing radiation. Flag-NAMPT proteins were precipitated from cell lysates 30 min after irradiation, washed twice with PBS and the binding affinity between NAMPT protein and PRPP was measured. **p < 0.01.

To determine the impact of AMPK-mediated S314 phosphorylation on NAMPT activity, we purified NAMPT protein from AMPK-dependent *in vitro* kinase assay, and found that S314 phosphorylation increased NAMPT enzymatic activiy by about five folds (figure 3*a*). By contrast, NAMPT S314A mutant protein showed a comparable activity as WT counterpart, regardless of whether it had been subjected to the *in vitro* kinase assay or not (figure 3*a*). In line with this, exposure to ionizing radiation, which resulted in robust NAMPT S314 phosphorylation, consolidated NAMPT activity in both HOK and HUVEC cells; this effect could be substantially abolished by S314A mutation (figure 3*b*).

Analysis of human NAMPT structure unveiled that S314 site is located within the catalytic domain (figure 3*c*). NAMPT condenses nicotinamide and PRPP to produce NMN [4]. Cocrystallization of NAMPT with PRPP (PDB code: 2E5C) or nicotinamide (PDB code: 2E5D) showed that S314 site is adjacent to PRPP, but far away from nicotinamide (figure 3*c*). It is documented that a phosphomimic of NAMPT histidine (H)247 was much more active than naive NAMPT protein, since it harboured a lower *K*_m_ value for PRPP (0.63 µM for NAMPT pH247; 7.2 µM for naive NAMPT) [23]. Strikingly, S314 is also close to H247, and these two sites and PRPP are situated in a triangular positioning in the catalytic domain. Therefore, we wandered whether phosphorylation of S314 likewise modulated the binding affinity between NAMPT and PRPP. To this end, NAMPT protein was precipitated after *in vitro* kinase assay, and incubated with [^32^P]-PRPP. As expected, NAMPT protein with S314 phosphorylation exhibited apparently stronger radioactive signal, compared to unphosphorylated control proteins, while similar effects were not observed in NAMPT S314A mutant protein (figure 3*e*). Consistently, S314A mutation or Compound C treatment also reduced enzymatic activity of NAMPT protein derived from irradiated cells (figure 3*f,g*). These results suggest that AMPK-dependent S314 phosphorylation activates NAMPT by facilitating NAMPT/PRPP association.

### 2.4. NAMPT S314 phosphorylation restores nuclear NAD, and facilitates DNA repair and cell survival under ionizing radiation

**Figure 4.**
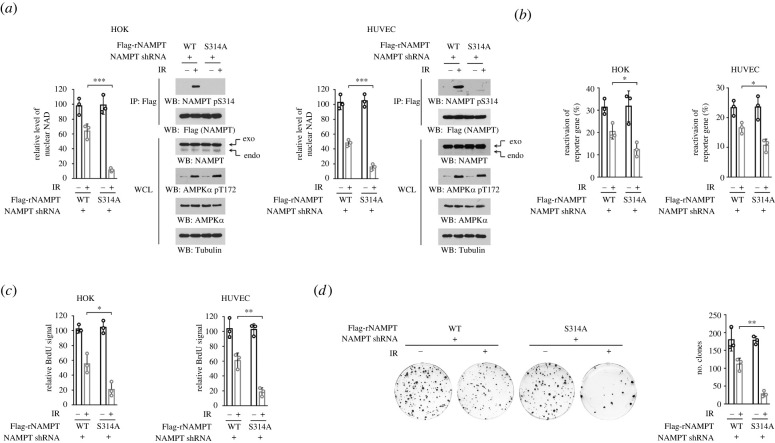
NAMPT S314 phosphorylation restores nuclear NAD, and facilitates DNA repair and cell survival. (*a*–*d*) Endogenous NAMPT-depleted HOK or HUVEC cells were stably expressed with shRNA-resistant (r) WT Flag-NAMPT or Flag-NAMPT S314A. Cells were treated with 10 Gy ionizing radiation. Nuclear NAD level was measured, and immunoprecipitates were performed using cell lysate 45 min after irradiation (*a*). The efficiency of the reactivation of reporter gene was measured 2 h after irradiation by luciferase assay (*b*). Cell viability was measured by BrdU incorporation assay 12 h after irradiation (*c*). HOK cell proliferation was measured by colony formation assay (*d*). IR, ionizing radiation; WCL, whole cell lysate; *p < 0.05; **p < 0.01; ***p < 0.001.

To explore the impact of AMPK-mediated NAMPT S314 phosphorylation on cell response to ionizing radiation, we knockdown the expression of endogenous NAMPT, and exogenously expressed Flag-tagged shRNA-resistant (r) WT NAMPT or NAMPT S314A mutant (figure 4*a*). Ionizing radiation-elicited DNA repair consumes NAD through protein poly(ADP-ribosyl)ation at DNA break sites [11]. Indeed, ionizing radiation caused 40–50% reduction in nuclear NAD levels in HOK and HUVEC cells (figure 4*a*). Reconstituted expression of NAMPT S314A mutant further shrank nuclear NAD pool, suggesting that NAMPT S314 phosphorylation contributed to maintaining NAD homeostasis (figure 4*a*). Additionally, NAMPT S314A mutation did not affect basal nuclear NAD level in the unirradiated cells.

NAD-dependent reactions are essential for DNA repair [13]. We then damaged a luciferase-based construct *in vitro* by irradiation, and transfected into HOK and HUVEC cells as a reporter gene to evaluate DNA repair capacity. In untreated cells, the DNA repair capacity was comparable regardless whether WT NAMPT and NAMPT S314A were expressed, and was more intense than irradiated cells (figure 4*b*). Upon ionizing radiation, a lowered luciferase signal was found in NAMPT S314A-expressed cells, hinting a further impaired DNA repair capacity in these mutant cells (figure 4*b*). Consistently, S314A mutation also palpably reduced cell viability and cell proliferation, shown by BrdU incorporation assay (figure 4*c*) and colony formation assay (figure 4*d*), respectively. These results suggest that AMPK-mediated NAMPT S314 phosphorylation restores nuclear NAD, and facilitates DNA repair and cell survival under ionizing radiation.

## 3. Discussion

RIOM is a common type of oral mucosal injury caused by radiotherapy that may lead to a significant adverse impact on the quality of life of patients and the continuity of cancer treatment. Epithelial and endothelial cells are radiosensitive [24,25]. The initial phase of radiotherapy causes direct and fatal DNA damage in epithelial and endothelial cells, which results in the release of reactive oxygen species, leading to the activation of multiple stress pathways and even cell death [10]. Therefore, oral epithelial HOK cells and endothelial HUVEC cells are widely used as *in vitro* models to study the radiation-induced oral damages and their underlying mechanisms [24,26,27]. Consistent with previous reports, we used HOK and HUVEC cells in this study to determine the impact of radiation on the NAD metabolism.

Serine/threonine kinase AMPK, a heterotrimer complex composed of catalytic *α* subunit and regulatory *β* and *γ* subunits, is a key modulator of signal transduction under multiple stressful conditions, including energy stress, DNA damage and hypoxia [28]. It is reported that DNA damages caused by etoposide, a potent double-strand break inducer [29], activates AMPK though modulating Ca^2+^/CaMKK2 signalling, which is independent of LKB1, the principal upstrean regulator of AMPK [30]. AMPK in turn regulates G2/M checkpoint and cell apoptosis by modulating p53 tumour suppressor and cyclin-dependent kinase inhibitor P21^waf/cip^ [31,32]. In addition, AMPK incurs p53-binding protein 1(53BP1) activity by directly phosphorylating 53BP1 at S1317, which recruits 53BP1 to the DNA damage site for effective DNA repair in a classical non-homologous end joining (C-NHEJ)-dependent manner [33]. Moreover, AMPK activity is found to modulate the radiosensitivity of cells through inhibition of the Akt-mTOR signalling pathway [18,31,34]. In this study, we illustrate that NAMPT S314, which matches the canonical AMPK phosphorylation consensus and is highly conserved during evolution, is a new substrate of AMPK. By *in vitro* kinase assay and autoradiography, we demonstrate that recombinate AMPK proteins introduced autoradiographic signal on the purified NAMPT protein, which was markedly blocked in the presence of Compound C. Since NAMPT couples ATP hydrolysis to NMN synthesis, formation of a high-energy phosphorylated intermediate NAMPT pH247 was previously observed [20]. Hence, in these *in vitro* kinase assays (figure 2), we used a boiled NAMPT protein, which did not show detectable autoradiographic signal after mixed with [γ-^32^P]-ATP, ruling out the potential interference caused by NAMPT autophosphorylation. Therefore, the current study expands the knowledge of AMPK-mediated signal transduction in the context of ionizing radiation. Considering that AMPK kinase consists of multiple subunits, further work is needed to distinguish the specific role of each subunit in the regulation of NAMPT.

The involvement of NAMPT and NAD metabolism in AMPK-mediated downstream transcriptional events has been widely documented. Under the conditions of fasting or caloric restriction, AMP-activated protein kinase (AMPK) is activated by lowered intracellular ATP availability, which in turn elevates NAMPT expression by boosting its gene transcription [35,36]. Upregulation of NAMPT results in an accelerated NAD production through salvage pathway and an increased activity of Sirtuin 1 (SIRT1), since lysine deacetylation by SIRT1 is coupled to the cleavage of NAD into nicotinamide and acetyl-ADP-ribose, and so the activities of SIRT1 are thus dependent on cellular NAD pool size. As a nuclear protein, SIRT1 deacetylates peroxisome proliferator-activated receptor gamma coactivator-1*α* (PGC-1*α*) to prompt mitochondrial biogenesis and ATP production, which can offset the harmful effects of energy stress [37]. Modulation of gene transcription takes a couple of hours to accumulate appreciable changes at protein level to affect cell phenotype. By contrast, repair of ionizing radiation-induced DNA double-strand breaks usually accomplished within 2 h, since a double-strand break is one of the most lethal types of DNA lesions, and delayed repair probably leads to cell death [38]. In the present data, AMPK activation was detected 15 min after exposure to ionizing radiation, evidenced by its active status marker T172 phosphorylation as well as the phosphorylation of its bona fide substrate ACC, which is consistent with previous reports [17,18]. Accordingly, phosphorylation of NAMPT S314 and increased cellular NAMPT activity could be detected within 30 min post-irradiation. Therefore, such post-translational modification-based mechanism reported in this study constitutes a novel route, by which cells can rapidly orchestrate NAD^+^ metabolism to support DNA repair in the context of ionizing radiation, presenting an important supplement to the established gene transcription-based regulatory pathway.

**Figure 5.**
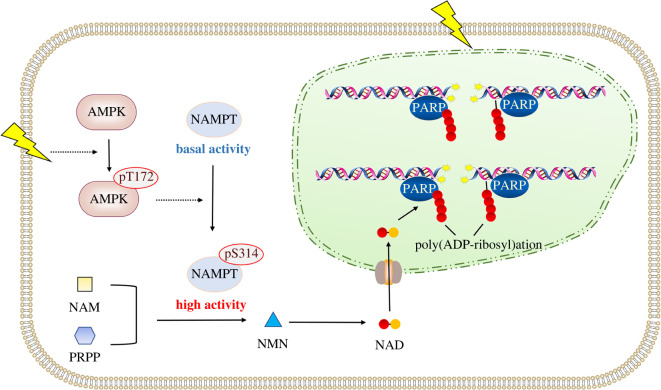
A schematic of AMPK-mediated NAMPT activation under ionizing radiation. Ionizing radiation causes rapid activation of AMPK, which in turn phosphorylates NAMPT S314 and enhances NAMPT enzymatic activity. NAMPT activation promotes NAD^+^ synthesis, thereby facilitating DNA repair and cell viability.

NAD^+^-dependent biochemistry reactions are vital for DNA damage repair and genome maintenance [39]. In this study, our findings illustrate a new stress-responsive mechanism under ionizing radiation. Ionizing radiation-elicited DNA damage signals to govern cellular NAD^+^ synthesis, through AMPK-mediated phosphorylation and activation of NAMPT (figure 5). Timely operation of this AMPK-guided metabolic cascade tunes NAD^+^ homeostasis and DNA repair, illuminating its potential value in the early prevention of radiation-induced oral mucositis.

## 4. Material and methods

### 4.1. Materials

Rabbit polyclonal antibody recognizing phosphorylated NAMPT pS314 was customized from Boer Biotechnology (Chengdu, China). To prepare antibody recognizing NAMPT pS314, rabbits were treated with peptide containing NAMPT pS314. Non-modified peptide immobilized on an affinity column was used to remove the antibodies recognizing non-phosphorylated NAMPT, and NAMPT pS314 peptide immobilized on an affinity column was used to associate with and isolate the antibodies. The eluted antibodies were then concentrated.

Antibodies recognizing NAMPT (#86634), ACC (#3676), ACC pS79 (#11818), AMPK*α* pT172 (#50081), and AMPK*α* (#5831) were obtained from Cell Signaling Technology. Antibodies recognizing Tubulin (ab7291), Flag (ab205606) and A769662 (ab120335) were purchased from Abcam. Anti-Flag agarose beads were obtained from Sigma. [γ-^32^P]-ATP was obtained from PerkinElmer (BLU002Z001MC). [^14^C]-nicotinamide was obtained from American Radiolabeled Chemicals (ARC 0794). Active AMPK proteins (14–840) were obtained from Sigma-Aldrich. KU55933 (S1092), NU7441 (S2638), AZD6738 (S7693), PD98059 (S1177), SP600125 (S1460) and Compound C (S7306) were obtained from Selleckchem.

### 4.2. Cell culture and irradiation

HOK cell was a gift provided by Dr J. S. Gutkind (National Institute of Dental and Craniofacial Research, MD, USA). HUVEC cells were obtained from ATCC. HOK cells were cultured in Dulbecco's modified Eagle's medium (Gibco; Thermo Fisher Scientific) supplemented with 10% fetal bovine serum, and HUVEC cells were cultured with F-12K medium. Ionizing radiation was performed with a ^137^Cs gamma-ray source at indicated doses.

### 4.3. Immunoprecipitation and immunoblot analysis

Immunoprecipitation and immunoblot analysis were performed following previous reports [40]. Cells were lysed with a buffer (0.1% SDS, 0.5 mM EDTA, 1% Triton X-100, 100 *μ*M sodium pyrophosphate,150 mM NaCl, 100 *μ*M PMSF, 100 *μ*M leupeptin, 1 *μ*M aprotinin, 1 mM dithiothreitol, 100 *μ*M sodium orthovanadate, 50 mM Tris–HCl [pH 7.5] and 1 mM sodium fluoride). Cell lysates were centrifuged at 11 000 g, and the supernatants were incubated with indicated antibodies overnight at 4°C. Then, the agarose beads were applied to the mixture for incubation for another 3 h. The protein-beads complexes were washed 3 times and then analysed by immunoblot.

### 4.4. DNA constructs and mutagenesis

Human NAMPT gene was cloned into pcDNA3.1/hygro(+)-Flag vector. QuikChange site-directed mutagenesis kit (Stratagene, La Jolla, CA) was used to prepare the mutants. shRNAs were prepared using the following sequences: NAMPT shRNA, TTA TTT CTA TTG GAA GAT G; control shRNA, GCT TCT AAC ACC GGA GGT CTT. shRNA-resistant (r) NAMPT was prepared by introducing four mutations (c334t, t336a, a339t, a342t) in the targeting site for NAMPT shRNA.

### 4.5. Purification of recombinant proteins

The DNA of WT Flag-NAMPT, Flag-NAMPT S314A and Flag-NAMPT T304A was cloned into pCold I vector (Takara Bio). Recombinant His/Flag-NAMPT and the mutant His/Flag-NAMPT proteins were expressed in BL21(DE3) bacteria as previously described [22]. Bacteria cells were cultured in Lysogeny Broth medium and expression of these proteins was induced by IPTG for 16 h at 30°C, followed by lysis via sonication.

To purify the His/Flag-NAMPT proteins, the lysed bacterial samples were transferred to a Ni-NTA column (GE Healthcare Life Sciences). The column was flushed with 20 mM imidazole and the protein was eluted with 250 mM imidazole. To remove contaminated proteins, the eluted samples were separated through a HiPrep 16/60 Sephacryl S-200 HR gel filtration column (GE Healthcare Life Sciences).

### 4.6. Measurement of NAMPT activity

The enzymatic activity of purified NAMPT protein was measured by using NAMPT Activity Assay Kit (Colorimetric) obtained from Abcam (ab221819), following manufacturer's instruction. Data were normalized to the level of input NAMPT protein.

Cellular NAMPT activity was measured following previous reports [41,42]. Briefly, after indicated treatment, cells were collected, sonicated in buffer containing 0.5 m NaCl, 20 mM Tris–HCl (pH 7.5), 10% glycerol and centrifuged. The supernatant was incubated with 0.4 mM PRPP and [^14^C]-nicotinamide (10 mCi mM^–1^) in a standard reaction mixture (30 µl) containing 50 mM Tris-HCl (pH 7.5), 10 mM MgCl_2_, 1 mM ATP and 2.5 mM dithiothreitol. After incubating at 37°C for 1 h, the reaction was terminated by boiling and the samples were deproteinized. The formed NMN was separated by thin layer chromatography on silica gel sheets (Merck) using an isobutyric acid-5% ammonium hydroxide-water mixture (66 : 10 : 19, v/v/v) as a solvent. The cellular NAMPT activity was determined according to the radioactivity of the samples. Data were normalized to cell number.

### 4.7. *In vitro* kinase assay

Kinase reactions were performed as described previously [16]. In brief, 10 ng purified recombinant AMPK proteins were incubated with 100 ng NAMPT in 25 µl of kinase buffer (50 mM Tris–HCl, pH 7.5, 100 mM KCl, 5 mM MgCl_2_, 1 mM Na_3_VO_4_, 50 µM DTT, 5% glycerol and 50 µM ATP) at 25°C for 1 h. 10 µCi [γ-^32^P]ATP and boiled NAMPT protein were used in the reaction system if autoradiography was used as the detection method. The reaction was terminated by boiling in sample buffer, and NAMPT proteins were precipitated and analysed by SDS-PAGE. The phosphorylation was detected by immunoblotting with indicated antibodies or by autoradiography.

### 4.8. Measurement of binding between NAMPT and PRPP

Immunoprecipitated NAMPT proteins from cell lysates or recombinate NAMPT proteins immobilized on beads were incubated with binding buffer (50 mM Tris–HCl (pH 7.5), 10 mM MgCl_2_ and 2.5 mM DTT) containing 0.4 mM PRPP and 20 µCi [^32^P]-PRPP at 30°C for 5 min. The protein-beads complexes were then washed with binding buffer twice, and the protein-associated radioactivity was detected by liquid scintillation counting. [^32^P]-PRPP was enzymatically synthesized using ribose 5-phosphate and [γ-^32^P]ATP, and isolated by ion-exchange chromatography as previously reported [43].

### 4.9. Measurement of DNA repair capacity

DNA repair capacity was measured following previous report [44]. Briefly, pGL2-CMV vector carrying a firefly luciferase gene was damaged *in vitro* by exposure to 20 Gy ionizing radiation, and used as a reporter gene. The cells were transfected with damaged or undamaged (positive control) pGL2-CMV vector, followed by indicated irradiation treatment 30 min after transfection. The cells were collected 72 h after the transfection, and the luciferase activity in the lysates was determined with a Promega luciferase assay system. The luciferase signal was normalized to total protein levels and represented as the percentage of positive control.

### 4.10. RT-PCR

Total RNA was isolated with reagent trizol, and then subjected to reverse transcription using first-strand cDNA synthesis for RT-PCR kit (Takara). The cDNA was analysed by PCR according to previously reported experimental protocols [24,45]. Primer sequences used for the indicated genes are as follows: NAMPT-F, 5′-GTA GTA ACC AAA GAG AAA ATC CAG GAA G-3′; NAMPT-R, 5′- GCT GTT ATG GTA CTG TGT TCT GCT G-3′; Actin-F, 5′-CAT GTA CGT TGC TAT CCA GGC-3′; Actin-R, 5′-CTC CTT AAT GTC ACG CAC GAT-3′.

### 4.11. Brdu incorporation assay

BrdU incorporation assay was performed by using BrdU Cell Proliferation ELISA Kit (colorimetric) obtained from Abcam (ab126556), following the manufacturer's instructions.

### 4.12. Colony formation assay

200 cells were seeded in 6-well plates. After indicated treatment, cells were continuously cultured for 12 days. The clones were fixed in methanol and stained with crystal violet solution. Clones with more than 50 cells were counted.

### 4.13. Measurement of nuclear NAD level

After indicated treatment, nucleus was isolated using a previously reported protocol, by which isolation of nucleus from cultured cells could be finished within 2 min [46]. The level of nuclear NAD was measured by using NAD/NADH Quantification Colorimetric Kit obtained from BioVision (K337-100), following the manufacturer's instructions.

### 4.14. Quantification and statistical analysis

Sample size was determined to be adequate based on the magnitude and consistency of measurable differences between groups in all experiments in this study. No randomization or blinding was done, and no sample was excluded from the analyses. Statistical analyses were performed using two-sided Student's *t*-test for comparison between two groups. All data represent the mean ± s.d. of at least three independent experiments/samples unless otherwise specified. Differences in means were considered statistically significant at *p* < 0.05. The Bonferroni correction was used for the multiple hypothesis correction (requiring *p* < 0.05/*N*, *N* indicates the number of comparisons), to avoid reporting false positive results. For every figure, statistical tests are justified as appropriate and the data met the assumptions of the tests. Finally, the variance between groups that were being statistically compared was similar.

## Data Availability

The data analysed during this study are included in this manuscript.
